# RECIL Versus Lugano for Treatment Response Assessment in FDG-Avid Non-Hodgkin Lymphomas: A Head-to-Head Comparison in 54 Patients

**DOI:** 10.3390/cancers12010009

**Published:** 2019-12-18

**Authors:** Dominik Berzaczy, Alexander Haug, Philipp B. Staber, Markus Raderer, Barbara Kiesewetter, Ulrich Jaeger, Christoph Kornauth, Ingrid Simonitsch-Klupp, Marius E. Mayerhoefer

**Affiliations:** 1 Department of Biomedical Imaging and Image-guided Therapy, Division of General and Pediatric Radiology, Medical University of Vienna, 1090 Vienna, Austria; marius.mayerhoefer@meduniwien.ac.at; 2 Department of Biomedical Imaging and Image-guided Therapy, Division of Nuclear Medicine, Medical University of Vienna, 1090 Vienna, Austria; alexander.haug@meduniwien.ac.at; 3 Department of Medicine I, Division of Hematology, Medical University of Vienna, 1090 Vienna, Austria; philipp.staber@meduniwien.ac.at (P.B.S.); ulrich.jaeger@meduniwien.ac.at (U.J.); 4 Department of Medicine I, Division of Oncology, Medical University of Vienna, 1090 Vienna, Austria; markus.raderer@meduniwien.ac.at (M.R.); barbara.kiesewetter@meduniwien.ac.at (B.K.); 5 Institute of Pathology, Medical University of Vienna, 1090 Vienna, Austria; christoph.kornauth@meduniwien.ac.at (C.K.); ingrid.simonitsch-klupp@meduniwien.ac.at (I.S.-K.); 6 Department of Radiology, Memorial Sloan-Kettering Cancer Center, NY 10065, USA

**Keywords:** lymphoma, FDG, PET/CT, treatment response

## Abstract

The response evaluation criteria in lymphoma (RECIL) classification for lymphoma treatment response assessment was introduced in 2017, but it has not yet been compared to the established Lugano classification. Also, the value of the provisional “minor response” (MiR) category of RECIL is unclear. In 54 patients with FDG-avid non-Hodgkin lymphomas (41 diffuse large B-cell lymphomas (DLBCL) and 13 follicular lymphomas), [^18^F]FDG-PET/CT-based response according to RECIL and Lugano was determined at interim and end-of-treatment (EOT) restaging. Rates of agreement and Cohen’s kappa (κ) coefficients were calculated. The relationship between RECIL and Lugano responses and 2-year complete remission (CR) status of DLBCL patients was determined. At interim restaging, MiR was observed in 14.8%, and at EOT, in 5.6% of patients. When MiR was recoded as partial remission, agreement between RECIL and Lugano was 83.3% at interim restaging (κ = 0.69), and 90.7% at EOT (κ = 0.79). 85.4%, of DLBCL patients with responding disease at interim restaging according to both RECIL and Lugano achieved 2-year CR status; whereas, at EOT, 82.9% of patients with responding disease according to Lugano, and 85.4% of patients with responding disease according to RECIL, achieved 2-year CR status. Thus, RECIL and Lugano classifications show comparable performance for treatment response assessment, and a similar association with 2-year CR status in FDG-avid lymphomas.

## 1. Introduction

The most widely used treatment response assessment system for lymphomas is the Lugano classification [1,2], which relies chiefly on the Deauville score for visual assessment of 2-deoxy-2-[^18^F]fluoro-D-glucose ([^18^F]FDG) uptake on positron emission tomography (PET) [3]. Morphological changes are irrelevant for Lugano-based response assessment in FDG-avid lymphomas such as diffuse large B-cell lymphoma (DLBCL) and follicular lymphoma (FL); whereas for lymphomas with variable FDG avidity (e.g., marginal zone lymphomas), bi-dimensional measurements of up to six target lesions are used [2].

Recently, a variation of the response criteria in solid tumors (RECIST) for lymphomas, termed RECIL, was proposed as an alternative to, or replacement of, the Lugano classification [4]. While the Deauville score is also an integral part of RECIL, there is more emphasis on morphological response on computed tomography (CT) than with Lugano, even for FDG-avid lymphomas. Assessment of morphological changes is easier with RECIL than with Lugano, as uni-dimensional measurements of just up to three target lesions are recommended.

So far, not a single comparative study between RECIL and Lugano has been performed in FDG-avid lymphomas using PET/CT or PET/MRI (magnetic resonance imaging). Moreover, the clinical significance of the novel response category introduced by RECIL—“minor response” (MiR)—has not been tested. Finally, it is unclear how the RECIL and Lugano classifications compare in terms of outcome prediction.

We therefore aimed to determine the level of agreement between RECIL and Lugano at interim and end-of-treatment (EOT) restaging in patients with FDG-avid non-Hodgkin lymphomas (NHL). We also investigated the incidence of RECIL’s MiR category, and its association with Lugano-based response. Finally, we compared the two response classifications in terms of their association with 2-year outcome.

## 2. Results

### 2.1. Patients

Out of 493 lymphoma patients that underwent [^18^F]FDG-PET/CT in the specified time period, 54 patients (24 women and 30 men; mean age, 58.2 ± 14.5 years)—41 with DLBCL and 13 with FL—met the criteria for participation in our study. Four patients had Ann Arbor stage I, 22 stage II, twelve stage III, and 16 patients stage IV disease (Table 1). Based on a total of 112 target lesions, the mean SUVmax at baseline was 13.2 ± 8.8, and the mean sum of the longest diameters (SLD) were 8.3 ± 4.7 cm. The mean SLD change relative to baseline was −32.5 ± 46.3% at interim restaging and −40.9 ± 59.6% at EOT.

### 2.2. Agreement between RECIL and Lugano

**Figure 1:**
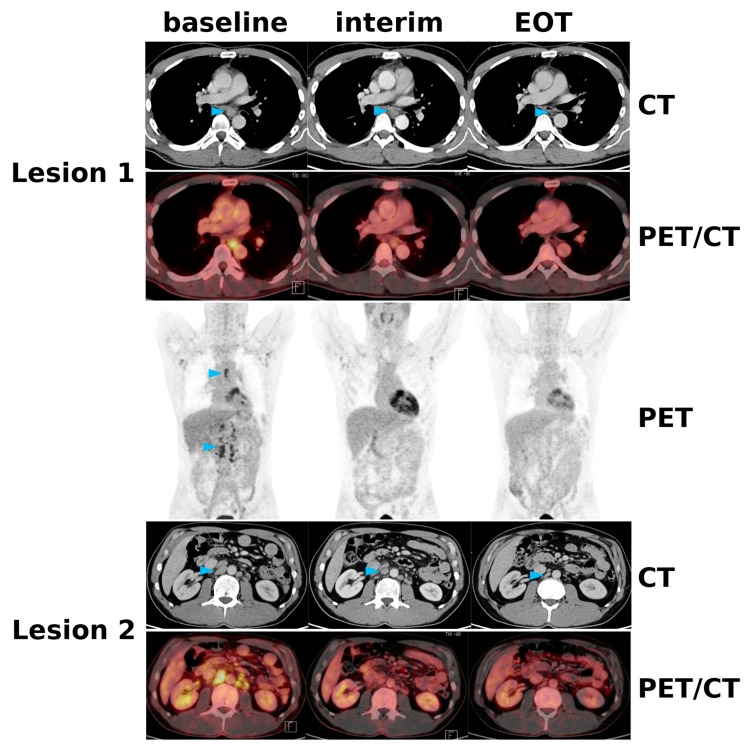
A 43-year-old patient with FL involving mediastinal and retroperitoneal (paraaortic) lymph nodes (blue arrowheads), as well as possible involvement of axillary and iliac lymph nodes. At interim restaging after three cycles of R-BENDA, the [^18^F]FDG uptake had decreased to Deauville ≤3 (i.e., not exceeding the liver uptake) in all nodal regions, which fulfills the Lugano criterion for CR. However, since the sum of the longest diameters (SLD) had only decreased by 24%, this represents MiR according to RECIL (Response Evaluation Criteria in Lymphoma) at interim restaging. At end-of-treatment (EOT), the SLD had finally decreased to 50%, consistent with CR according to both RECIL and Lugano.

At interim restaging, MiR was observed in 8/54 patients (14.8%), all of which showed responding disease (two partial remissions (PR) and six complete remissions (CR)), according to Lugano (Table S1). Minimal response (MiR) at EOT was observed in 3/54 patients (5.6%); one patient each with CR, PR, and SD, according to Lugano. Seven patients (87.5%) with MiR at interim restaging showed PR or CR at EOT according to Lugano (Figure 1), whereas 1/9 patients (12.5%) showed progressive disease (PD) at EOT (Table S2).

**Figure 2:**
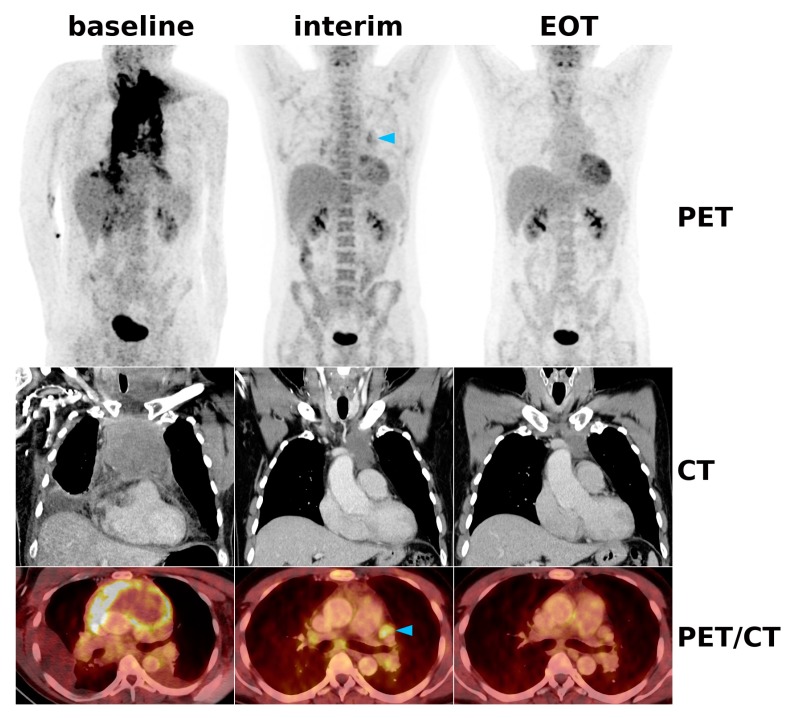
A 44-year-old patient with DLBCL involving the mediastinal, hilar, and lower neck lymph nodes. At interim restaging after three cycles of R-CHOP, the initially strong [18F]FDG uptake had dramatically decreased in all nodal regions, with only some hilar and mediastinal lymph nodes (blue arrowhead) still showing slightly higher uptake than the liver (i.e., Deauville 4 with reduced uptake), which fulfills the Lugano criterion for PR. Since the size of the lymph nodes, including the dominant bulk in the mediastinum, had clearly decreased by >30%, this also represented PR according to RECIL. At EOT, the [18F]FDG uptake was now lower than that of the liver and mediastinal blood pool (i.e., Deauville 2), fulfilling the CR criteria of both Lugano and RECIL.

When MiR was recoded as PR, agreement between RECIL and Lugano was 83.3% at interim restaging (45/54 patients; Cohen’s kappa (κ) = 0.69, *p* < 0.0001), and 90.7% at EOT (49/54 patients; κ = 0.79, *p* < 0.0001) (Figure 2). Of the nine cases with disagreement between the two classifications at interim restaging, RECIL suggested a poorer response than Lugano in 67% (6/9); whereas at EOT, RECIL suggested a more favorable response than Lugano in 80% (4/5) of cases with disagreement between the two classifications, respectively.

When MiR was recoded as SD, agreement between RECIL and Lugano at interim restaging was 79.6% (43/54 patients; κ = 0.63, *p* < 0.0001), and 90.7% at EOT (49/54 patients; κ = 0.79, *p* < 0.0001). Of the eleven cases with disagreement between the two classifications at interim restaging, RECIL suggested a poorer response than Lugano in 72.7% (8/11); whereas at EOT, RECIL suggested a more favorable response than Lugano, in 60% (3/5) of the cases with disagreement between the two classifications, respectively.

### 2.3. Association with 2-Year Complete Remission (CR) Status

**Figure 3:**
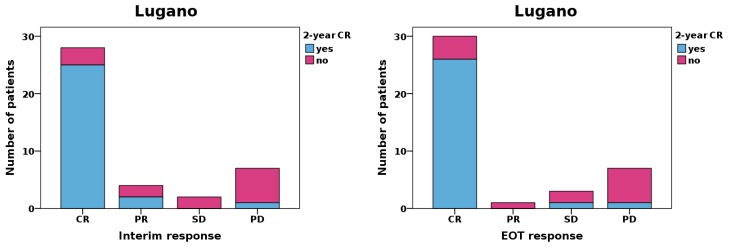

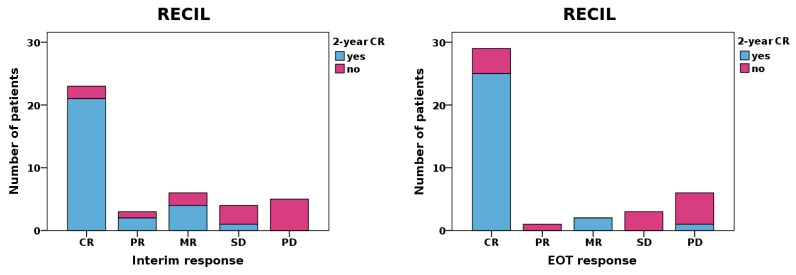
Relationship between RECIL and Lugano responses and 2-year CR status. Patients with CR, PR, MR, SD, and PD responses according to RECIL at interim restaging achieved 2-year CR status in 91.3%, 66.7%, 66.7%, 25.0%, and 0.0% of cases; and with the respective responses at EOT, in 86.2%, 0.0%, 100.0%, 0.0%, and 16.7% of cases. By comparison, patients with CR, PR, SD, and PD responses according to Lugano at interim restaging achieved 2-year CR status in 89.3%, 50.0%, 0.0%, and 14.3% of cases; and with the respective responses at EOT, in 86.7%, 0.0%, 33.3%, and 14.3% of cases. Overall, patients with MiR achieved better outcomes than patients with SD.

Due to the low number of FL patients, the Lugano and RECIL responses (Table S3) were only tested for their association with 2-year outcome in the 41 DLBCL patients. In this group, 28/41 patients (68.3%) achieved 2-year CR status, whereas 13/41 patients (31.7%) showed residual/recurrent disease; no deaths occurred within the observation period. The relationship between 2-year CR status and interim and EOT responses according to RECIL and Lugano are shown in Figure 3.

85.4%, of DLBCL patients with responding disease at interim restaging according to both RECIL-1 and Lugano, and 80.5% according to RECIL-2 achieved 2-year CR status. Four of six patients (66.7%) with MiR at interim restaging were in CR after two years.

By contrast, 82.9% of DLBCL patients with responding disease at EOT according to Lugano, 85.4% of patients with responding disease according to RECIL-1, and 80.5% according to RECIL-2 achieved 2-year CR status. Both of the patients (100%) with MiR at EOT restaging were in CR after two years.

### 2.4. Reading Times for RECIL and Lugano

For RECIL, mean reading times for raters 1 and 2 were 3.4 ± 0.7 min and 3.8 ± 0.6 min, respectively, whereas for Lugano, mean reading times were 1.8 ± 0.3 min and 1.9 ± 0.2 min, respectively.

## 3. Discussion

Our results suggest that, at EOT, agreement between RECIL and Lugano is very good (91%, regardless of whether MiR was recoded as SD or PR), whereas at interim restaging, agreement may be lower, but is still substantial (~80%). Minor response according to RECIL was a rare finding at EOT (5–6% of patients), whereas it was, with 15%, quite common at interim restaging. RECIL and Lugano interim responses show a comparable association with 2-year CR status in DLBCL, whereas the RECIL response at EOT showed a slightly stronger association with 2-year CR status than the respective Lugano-based response.

RECIL was developed to provide a higher level of structural consistency with RECIST, the response assessment system used in most clinical trials; and to provide criteria that are easier to use than the Lugano criteria. With Lugano, morphological response relies on bi-dimensional measurements of up to six target lesions, which is time-consuming. Contrary, RECIL recommends uni-dimensional measurements of just up to three target lesions. In 2983 lymphoma patients from ten multi-centric clinical trials, a high correlation between uni- and bi-dimensional measurements, and no relevant effect on the response category to which patients were assigned, were observed [4].

However, since the majority of lymphomas are FDG-avid [5], and should therefore undergo [^18^F]FDG-PET/CT for response assessment according to the International Conference for Malignant Lymphomas [3], the morphological Lugano criteria are rarely applied, except in areas without access to PET/CT. For FDG-avid lymphomas, the [^18^F]FDG-PET/CT-based Lugano criteria are less complex than RECIL, because the lesions’ uptake relative to the liver uptake is the main criterion—contrary to RECIL, no size measurements are required. On the other hand, PR, SD, and PD within the Lugano criteria rely on the rather subjective terms “decreasing”, “unchanged”, or “increasing” [^18^F]FDG uptake, for which no clear quantitative definitions exist—this represents a clear limitation.

The clinical value of minor response, the provisional RECIL category, is unproven. We observed MiR mainly at interim restaging, where it was the second-most common response type after CR. Seventy-five percent of patients with MiR showed CR by Lugano at interim restaging, and none showed PD. However, while most patients with MiR at interim restaging also showed CR or PR at EOT according to Lugano—suggesting that MiR generally indicates a favorable outcome—one patient showed progression due to new lesion development. Response according to RECIL was generally poorer at interim restaging (especially when MiR was recoded as SD), and more favorable at EOT (especially when MiR was recoded as PR), compared to Lugano. Therefore, when agreement with Lugano is desirable, MiR should be recoded as PR for interim, and as SD for EOT restaging.

With regard to 2-year CR status, RECIL and Lugano responses at interim restaging showed an identical association when MiR was—as suggested by the International Working Group—regarded as responding disease. Notably, using this approach, RECIL at EOT was even more strongly associated with 2-year CR status than Lugano at EOT, albeit only slightly. These results support data provided by previous studies in DCBCL and its variant, primary mediastinal B-cell lymphoma, which suggested a prognostic value of PET-based response at interim restaging or EOT [6,7].

Study limitations included the retrospective design, the moderate sample size, and inclusion of only the two most common NHL subtypes, DLBCL and FL—no patients with Hodgkin lymphoma or other more commonly seen lymphomas such as mantle cell lymphoma were evaluated. We chose 2-year outcome as our clinical endpoint, because 5-year outcome data were only available in a small fraction of our patients. Furthermore, we used CR status instead of survival, because in view of our moderate size and the rather short observation period, the use of survival rates was regarded as not meaningful; this assumption was confirmed by the fact that no deaths occurred in the DLBCL group within the 2-year interval. Finally, in DLBCL patients, we did not distinguish between low- and high-risk patients, nor did we take into account mutational status that is possibly related to therapy resistance [8], because this was of no relevance for the present study, whose goal it was to compare the two PET-based response assessment systems, Lugano and RECIL in the same patient cohort.

## 4. Materials and Methods

### 4.1. Patients and Design

Patients with histology-proven, treatment-naïve DLBCL or FL, who had undergone [^18^F]FDG-PET/CT as part of the routine clinical workup between January 2016 and October 2017 for baseline staging, then interim restaging after three immunochemotherapy cycles and at EOT after six cycles of the same treatment were eligible for inclusion in this retrospective study that was conducted at a single tertiary care center. For DLBCL patients, treatment was either R-CHOP (rituximab, cyclophosphamide, doxorubicin, vincristine, and prednisone) or dose-adjusted R-EPOCH (rituximab, etoposide, doxorubicin, vincristine, cyclophosphamide, and prednisone), both of which are considered “optimal” treatments [9]; whereas for FL patients, the treatment was R-BENDA (rituximab and bendamustine) in all cases. The study was approved by the Ethics Committee of the Medical University of Vienna (protocol code: 1701/2018); informed consent was waived due to the retrospective design. Patients with blood glucose levels >150 mg/dL at any PET/CT examination were excluded. PFS (progression-free survival) was obtained from the electronic medical records of the hospital information system, using the oncologists’ assessment, which was based on physical examination and interventional, laboratory, and imaging tests.

### 4.2. Imaging Protocols

[^18^F]FDG-PET/CT was performed using a 64-row multi-detector, hybrid PET/CT system (Biograph TruePoint TrueView 64; Siemens, Erlangen, Germany). The PET system offers an axial field-of-view of 216 mm, a sensitivity of 7.6 cps/kBq, and a transaxial resolution of 4–5 mm (measured according to NEMA NU2 protocol). PET was performed 60 min after an intravenous administration of 3MBq/kg of [^18^F]FDG, at 4-min/bed position; PET images were reconstructed using the point-spread function (PSF)-based reconstruction algorithm TrueX, with four iterations and 21 subsets, 5-mm slice thickness, and a 168 × 168 matrix size. Venous-phase contrast-enhanced CT was used for attenuation correction, and was performed after the intravenous injection of 90–120 mL of a tri-iodinated, non-ionic contrast medium at a rate of 4 mL/s, with a reference tube current of 230 mAs (with tube current modulation), a tube voltage of 120 kVp, a collimation of 64 × 0.6 mm, a 5-mm slice thickness with a 3-mm increment, and a 512 × 512 matrix.

### 4.3. Image Analysis

[^18^F]FDG-PET/CT was analyzed, in consensus, by a single rater team, consisting of a board-certified radiologist and a board-certified nuclear medicine physician. Baseline staging was performed according to the Ann Arbor classification [10], taking into account the results of iliac crest bone marrow biopsy, where necessary (i.e., for all FL cases, and for DLBCL cases with a negative [^18^F]FDG-PET, according to guidelines [1]). Stage I was defined as involvement of a single anatomic site (i.e., single lymph node region or extranodal site with a single lesion); stage II as involvement of two or more anatomic sites on the same side of the diaphragm (e.g., two lymph node regions); stage III as involvement of anatomic sites on both sides of the diaphragm; and stage IV as multifocal or diffuse extranodal involvement.

Blinded to follow-up scans, the up to three largest nodal or extranodal lymphoma manifestations (minimum long-axis diameters, 1.5 and 1.0 cm, respectively) were identified at baseline in accordance with RECIL, and their long-axis diameters were measured on CT. For PET-based treatment response assessment at interim and EOT restaging, the Deauville score that reflects the post-therapeutic FDG uptake in lesions relative to that of pre-defined reference tissues was used [3].

For Lugano, post-therapeutic FDG uptake not exceeding that of the liver (Deauville 1–3) was rated as complete remission (CR); uptake exceeding that of the liver (Deauville 4–5), but with reduced uptake relative to baseline, was rated as partial remission (PR); Deauville 4–5 with unchanged uptake was rated as stable disease (SD); and Deauville 4–5 with increased uptake, and/or appearance of new FDG-avid lesions, was rated as progressive disease (PD).

For RECIL, a ≥30% decrease in SLD (sum of the longest diameters of the target lesions) with Deauville 1–3 was required for CR, and a ≥30% SLD decrease with Deauville 4–5 for PR. For SD, an SLD decrease <10% or an increase ≤20%, with any Deauville score, was required; whereas for PD, an >20% SLD increase with any Deauville score, or appearance of new PET-positive lesions. For RECIL’s new MiR (minor response), an SLD decrease ≥10% but <30%, with any Deauville score, was required [4].

To compare the reading times required for RECIL and Lugano, all cases were re-read independently under stop-clock conditions, by two board-certified reviewers, each with >5 years of PET/CT experience, separately for RECIL and Lugano with one week between the evaluations, and reading times (in minutes) were recorded.

### 4.4. Statistical Analysis

Apart from descriptive data, rates of agreement between RECIL and Lugano, and corresponding Cohen’s kappa (κ) coefficients [11] were calculated, separately for interim and EOT restaging. This step was performed twice: Once with the MiR category of RECIL recoded as PR, and once with MiR recoded as SD. Agreement between RECIL at interim restaging and EOT responses was also assessed. Percentages of patients achieving 2-year CR status were calculated for RECIL and Lugano responses at interim and EOT. For the latter, both RECIL and Lugano responses were categorized as “response” or “no response”. For Lugano, “response” comprised PR and CR, and “no response” SD and PD. For RECIL, two variations were used:

- RECIL-1: “response” (MiR, PR, or CR) vs. “no response” (SD or PD); and
- RECIL-2: “response” (PR or CR) vs. “no or minor response” (MiR, SD, or PD).

Pearson chi-square tests were used for all group comparisons. The level of significance was *p* ≤ 0.05 for all tests, which were performed using SPSS 24.0 (IBM, Armonk, NY, USA).

## 5. Conclusions

This RECIL and Lugano classifications yielded comparable information about lymphoma treatment response in about 90% of patients at EOT, with RECIL providing a slightly more optimistic assessment of treatment response. Minor response, the novel and provisional RECIL category, appears to be a rare finding at EOT, but a common one at interim restaging, where it generally indicates responding disease. While our results suggest that RECIL and Lugano show a similar association with 2-year CR status, larger studies with longer observation periods and additional clinical endpoints are required to confirm this notion. While the Lugano classification is slightly less time-consuming, neither of the two classifications appears to be clearly superior to the other, and thus, no clear recommendation for either of the two can be made.

## Figures and Tables

**Table 1:** Patient demographics by lymphoma subtype.

Demographics:	Diffuse Large B-Cell Lymphoma (DLBCL)	Follicular Lymphoma (FL)
Age (mean ± SD)	55.5 ± 17.5	61.8 ± 11.1
Sex (m/f)	25/16 (61%/39%)	5/8 (38.5%/61.5%)
Ann Arbor I	2	2
Ann Arbor II	18	4
Ann Arbor III	9	3
Ann Arbor IV	12	4
2-year complete remission (CR) status	28/41 (68.3%)	10/13 (76.9%)

## Supplementary Materials

The following are available online at https://www.mdpi.com/2072-6694/12/1/9/s1. Table S1: Treatment response: Lugano versus RECIL in 54 patients, Table S2: Relationship between RECIL-based interim response and Lugano-based EOT response, Table S3: Lugano versus RECIL in 41 DLBCL patients.
