# Supplementary material for: RECIL Versus Lugano for Treatment Response Assessment in FDG-Avid Non-Hodgkin Lymphomas: A Head-to-Head Comparison in 54 Patients
Source: Cancers (Basel). 2019 Dec 18;12(1):9. doi: 10.3390/cancers12010009 (PMC7016710; doi:10.3390/cancers12010009)
Supplement: Supplementary file 1 [file cancers-12-00009-s001.pdf]

cancers

Supplementary Materials

Table S1. Treatment response: Lugano versus RECIL in 54 patients.

**Interim restaging:**		RECIL					
		CR	PR	MiR	SD	PD	Total
**Lugano**	CR	32	0	6	0	0	38
	PR	0	3	2	0	0	5
	SD	0	0	0	2	0	3
	PD	0	0	0	3	5	8
	Total	32	3	8	6	5	54
**End of treatment:**		**RECIL**					
		CR	PR	MiR	SD	PD	Total
**Lugano**	CR	39	0	1	0	0	40
	PR	0	1	1	0	0	2
	SD	0	0	1	2	0	3
	PD	0	0	0	3	6	9
	Total	39	1	3	5	6	54

CR, complete remission; PR, partial remission; MiR, minor response; SD, stable disease; PD, progressive disease

Table S2. Relationship between RECIL-based interim response and Lugano-based EOT response.

		**RECIL interim**					
		CR	PR	MiR	SD	PD	Total
**Lugano EOT**	CR	32	2	5	0	1	40
	PR	0	0	2	0	0	2
	SD	0	0	0	3	0	3
	PD	0	1	1	3	4	9
	Total	32	3	8	6	5	54

CR, complete remission; PR, partial remission; MiR, minor response; SD, stable disease; PD, progressive disease

Table S3. Lugano versus RECIL in 41 DLBCL patients.

**Interim restaging:**		**RECIL**					
		CR	PR	MiR	SD	PD	Total
**Lugano**	CR	23	0	5	0	0	28
	PR	0	3	1	0	0	4
	SD	0	0	0	2	0	2
	PD	0	0	0	2	5	7
	Total	23	3	6	4	5	41
**End of treatment:**		**RECIL**					
		CR	PR	MiR	SD	PD	Total
**Lugano**	CR	29	0	1	0	0	30
	PR	0	1	0	0	0	1
	SD	0	0	1	2	0	3
	PD	0	0	0	1	6	7
	Total	29	1	2	3	6	41

CR, complete remission; PR, partial remission; MiR, minor response; SD, stable disease; PD, progressive disease
